# Cardiac Autonomic Dysfunction in Obstructive Sleep Apnea: The Hidden Role of Vitamin D Deficiency

**DOI:** 10.1111/jebm.70071

**Published:** 2025-09-17

**Authors:** Huai Heng Loh, Siow Phing Tay, Ai Jiun Koa, Mei Ching Yong, Asri Said, Chee Shee Chai, Natasya Marliana Abdul Malik, Anselm Ting Su, Bonnie Bao Chee Tang, Florence Hui Sieng Tan, Norlela Sukor

**Affiliations:** ^1^ Department of Medicine Faculty of Medicine Universiti Kebangsaan Malaysia Kuala Lumpur Malaysia; ^2^ Faculty of Medicine and Health Sciences Universiti Malaysia Sarawak Sarawak Malaysia; ^3^ Department of Medicine Sarawak General Hospital Ministry of Health Sarawak Malaysia; ^4^ Medical Division Sunway Medical Center Damansara Selangor Malaysia; ^5^ Department of Medicine Hospital Canselor Tuanku Muhriz Kuala Lumpur Malaysia

1

Obstructive sleep apnea (OSA) is a common sleep‐breathing disorder, affecting an estimated 9–38% of adults worldwide, with prevalence increasing in parallel with rising obesity rates and aging population [1]. While men are still more likely to be diagnosed with OSA, this condition is becoming more common in women [2]. Coronary artery disease (CAD), a common complication of OSA, is driven by a combination of intermittent hypoxia, metabolic dysfunction, and systemic inflammation, which together accelerate atherosclerosis [3]. This underscores the importance of identifying modifiable factors to mitigate cardiovascular risks in these patients. A key mechanism linking OSA to heightened cardiovascular risks in CAD is cardiac autonomic dysfunction, characterized by increased sympathetic activity and reduced parasympathetic tone. These alterations can be assessed through heart rate variability (HRV), a well‐established predictor of adverse cardiovascular outcomes.

Patients with OSA often exhibit lower serum 25‐hydoxyvitamin D [25(OH)D] levels, with a high prevalence of vitamin D deficiency (VDD) compared to those without OSA [4]. OSA and VDD share overlapping risk factors, with interplay between obesity, hypoxia, reduced vitamin D absorption, inflammation, and effects on upper airway muscle function [4]. VDD is linked to higher cardiovascular risk and mortality and may be associated with poorer HRV [5, 6], although evidence remains limited.

This study aimed to investigate the role of vitamin D in cardiovascular autonomic function among OSA patients, addressing a critical gap in understanding the interplay between VDD, OSA, and cardiovascular risks. Given the contribution of systemic inflammation and endothelial dysfunction to cardiovascular risks in OSA, we also assessed high‐sensitivity C‐reactive protein (hsCRP) and brachial artery flow‐mediated dilatation (BAFMD) as secondary endpoints, providing complementary insights alongside cardiac autonomic function. The study was conducted in accordance with the principles outlined in the Declaration of Helsinki. Ethical approval was obtained from the Medical Research Ethical Committee of Malaysia (NMRR‐21‐1472‐60812) and UNIMAS Medical Ethics Committee (UNIMAS/TNC(PI)/09‐65/01). All patients who fulfilled study criteria were recruited after informed consent obtained. This study was part of a broader research effort, *Cardiovascular Impacts of RAAS and Vitamin D in Obstructive Sleep Apnea (CARD‐OSA)*, which sought to explore the complex roles of renin‐angiotensin‐aldosterone system and vitamin D in OSA and their broader implications for patient health.

Designed as a cross‐sectional study, it was conducted at a tertiary sleep center from June 2022 to May 2024. Briefly, patients aged ≥18 years with a body mass index (BMI) ≥27.5 kg/m^2^, and confirmed OSA were recruited. The BMI cut‐off used, following WHO Asia‐Pacific criteria for obesity, aimed to focus on obese individuals, who comprise a high‐risk subgroup for both OSA and VDD. Those who were on vitamin D and calcium supplements, had chronic kidney disease, malignancy, cardiac rhythm abnormalities, congestive heart failure, uncontrolled thyroid or parathyroid disease, and pregnancy were excluded from the study.

Although the study participants were not formally screened for CAD, data on co‐morbidities from medical records were documented. All participants underwent assessments for anthropometry, serum 25(OH)D, metabolic parameters, hsCRP, and HRV. Cardiac autonomic function was assessed using Polar H10 strap sensor over 3 min for short‐term HRV data, and 24‐h Holter monitoring for long‐term HRV analysis. Ultrasound of BAFMD was assessed as per protocol [7]. Potential confounders in regression analysis were selected based on established links to HRV and vitamin D status in prior literature and their clinical relevance in OSA.

The HRV parameters included for analysis and their normal values and ranges are displayed in Tables S1 and S2 respectively. OSA severity was categorized into mild (apnoea‐hypopnoea index, AHI 5 to <15/h), moderate (AHI 15 to <30/h), and severe (AHI ≥ 30/h). Vitamin D was categorized based on serum 25(OH)D levels, that is, <20 ng/mL as VDD, and ≥20 ng/mL as non‐VDD (nVDD). Normal endothelial function assessed by BAFMD was defined as ≥7.1%, the optimal cut‐off for distinguishing individuals with cardiovascular risks [8].

A total of 797 patients suspected of OSA were screened and 204 (mean age 43.4, 49% males) were recruited (Figure S1). Most patients had severe OSA, with median AHI of 39.0/h (range of AHI 30.1–125.9/h). Demographic data, co‐morbidities, metabolic profile, cardiovascular risk markers, and HRV parameters of the study participants are displayed in Table S3. Comparing to those in nVDD group, patients with VDD were younger, consisted mainly of Malay ethnicity and female participants, with lower prevalence of dyslipidemia but higher BMI. Otherwise, there was no significant difference in other co‐morbidities and metabolic parameters. Patients with VDD had higher hsCRP (Table S3), and poorer HRV (Table 1) compared with the nVDD group. However, there was no difference in BAFMD.

**TABLE 1:** Heart rate variability parameters of study participants.

	All (*n* = 204)	VDD (*n* = 118)	nVDD (*n* = 86)	*p* value	Mean difference (95% CI)
Holter parameters					
Log ASDNN, ms	1.64 ± 0.16	1.62 ± 0.16	1.68 ± 0.15	0.011	−0.057 (–0.101, –0.013)
Log SDANN, ms	2.00 ± 0.17	1.94 ± 0.17	2.01 ± 0.15	0.002	−0.060 (–0.101, –0.013)
Log SDNN, ms	2.02 ± 0.15	1.99 ± 0.16	2.06 ± 0.14	0.001	−0.070 (–0.112, –0.028)
Polar H10 parameters					
Log SDNN, ms	1.27 ± 0.32	1.25 ± 0.36	1.29 ± 0.26	0.386	−0.040 (–0.132, 0.051)
Log RMSSD, ms	1.21 ± 0.37	1.17 ± 0.41	1.27 ± 0.30	0.059	−0.100 (–0.203, 0.004)
PNS index	−1.26 ± 1.11	−1.45 ± 1.12	−1.00 ± 1.04	0.005	−0.447 (–0.757, –0.138)
SNS index	3.17 ± 3.01	3.68 ± 3.25	2.47 ± 2.48	0.005	1.210 (0.370, 2.050)
Log stress index	1.29 ± 0.24	1.31 ± 0.27	1.27 ± 0.21	0.232	0.042 (–0.027, 0.111)

*Note*: Numerical variables are presented as the mean ± standard deviation.

Abbreviations: ASDNN, mean of the standard deviations of all NN intervals for all 5‐min segments of entire recording; CI, confidence interval; nVDD, non‐vitamin D deficiency; PNS, parasympathetic nervous system; SNS, sympathetic nervous system; RMSSD, square root of the mean of the squares of successive NN interval differences; SDANN, standard deviation of 5‐min average NN intervals; SDNN, standard deviation of the NN intervals; VDD, vitamin D deficiency.

Pearson's correlation showed serum 25(OH)D levels were negatively associated with sympathetic nervous system (SNS) index (*r* = –0.185, *p* = –0.009) and stress index (*r* = –0.145, *p* = 0.041) (Table S4). This association remained significant even after adjusting for confounding factors, as illustrated in the multivariate linear regression analysis (Table S5). The best‐fitting models with the highest adjusted *R* ^2^ were chosen leading to the differences in variables between the two models.

Our study revealed two key findings: first, OSA patients with VDD exhibit lower HRV and higher hsCRP compared to nVDD patients; second, lower vitamin D concentrations are correlated with poorer HRV, particularly in terms of heightened SNS activation, as evidenced by significantly higher SNS and stress indices. These findings underscore the intricate interplay between vitamin D status and cardiac autonomic function in OSA patients.

The association between OSA and VDD has been well established, with a bi‐directional relationship detailed in previous literature [4]. Although evidence remains inconsistent whether hypovitaminosis D directly contributes to adverse cardiovascular outcomes, several potential pathways have been proposed [9]. These mechanisms include oxidative stress and inflammation, which promote vascular injury, reflected by the higher hsCRP levels observed in the VDD group. Endothelial dysfunction—characterized by impaired nitric oxide availability and vascular reactivity—was not evident in our cohort, whereas dysregulation of cardiac autonomic control was apparent, with poorer HRV indices in VDD patients indicating a shift toward sympathetic predominance and reduced parasympathetic activity.

Vitamin D influences cardiac tissues and modulates cardiac contractility via calcium metabolism and VDR activity. Low vitamin D levels have also been implicated in structural and ionic channel remodeling, leading to prolonged repolarization intervals and abnormal parasympathetic activity [10]. This aligns with our findings, where serum 25(OH)D levels were significantly associated with HRV parameters, particularly the SNS and stress indices, even after adjusting for confounders, although the low adjusted *R* ^2^ indicates that the model explains a modest proportion of the variance in HRV. These results suggest that hypovitaminosis D, alongside OSA severity, may partly contribute to elevated cardiovascular risks in OSA patients by increasing SNS activity and disrupting autonomic balance.

HRV, a non‐invasive measure of cardiac autonomic function, reflects fluctuations in SNS and PNS activity. Abnormal HRV is linked to heightened risks of cardiovascular disease and mortality. Reduced HRV, indicative of heightened SNS activity or diminished PNS activity, has been linked to adverse outcomes, including malignant arrhythmias and sudden cardiac death [11]. Short‐term HRV assessment, such as those performed in our study, has gained popularity for its ability to predict mortality and ease of artifact handling compared to 24‐h recordings, which are more susceptible to inter‐individual variability from daily activities [12]. The use of Polar H10 device in our study ensured precise measurement of these indices, further strengthening the reliability of our findings.

Despite the robustness of our study, several limitations should be noted. First, the cross‐sectional design of our study precludes determination of causality between VDD and cardiovascular risks in OSA patients. Second, genetic polymorphisms related to vitamin D, such as those involving vitamin D binding protein and vitamin D receptors, were not investigated due to resource and budget constraints. These genetic factors may further elucidate individual variability in the relationship between vitamin D status and cardiac autonomic function. Third, as our cohort comprised a higher proportion of females (51%) and middle‐aged adults, the findings may not be generalizable to the broader OSA population. This may reflect local referral patterns and healthcare‐seeking behaviour among women and the middle‐aged individuals [13, 14]. In addition, men with OSA and older patients in our setting may have had a greater burden of established cardiovascular complications or advanced organ dysfunction, leading to their exclusion based on our study criteria. Besides, hsCRP and BAFMD measured in our study provide only a partial representation of inflammatory status and endothelial function. The absence of other markers including interleukin‐6, tumor necrosis factor‐6, and measures of microvascular endothelial function limits a more comprehensive assessment of the pathways linking VDD, inflammation, endothelial health, and cardiac autonomic dysfunction.

Despite these limitations, this is, to the best of our knowledge, the first study to explore the role of vitamin D in cardiac autonomic function among OSA patients. By excluding individuals receiving vitamin D supplementation, we minimized confounders and ensured that our results more accurately reflect the natural impact of vitamin D status on cardiac autonomic function in this population.

Moving forward, longitudinal studies are needed to confirm these findings and to evaluate the potential role of vitamin D supplementation in mitigating cardiac autonomic dysfunction and cardiovascular risks in this population. Future research should explore optimal vitamin D thresholds for cardiovascular protection and the impact of supplementation on HRV over time.

In conclusion, VDD may contribute to the autonomic dysfunction through mechanisms that partially account for the increased cardiovascular risks observed in OSA patients. This highlights the importance of VDD screening in this high‐risk population, potentially guiding risk stratification and integrative management strategies.

## Conflicts of Interest

The authors declare no conflicts of interest.

## Supporting information




**Figure S1**: The recruitment of patients.
**Table S1**: Mean ± SD and range of short‐ and long‐term HRV parameters summarized from available cross‐sectional studies data.
**Table S2**: Parasympathetic nervous system and sympathetic nervous system zones interpretation and stress index normal range (from Kubios user guide).
**Table S3**: Characteristics of study participants.
**Table S4**: Correlation between serum 25(OH)D and heart rate variability parameters.
**Table S5**: Multivariate analysis between heart rate variability parameters and 25(OH)D and covariates in patients with OSA.
