# Supplementary material for: Cardiac Autonomic Dysfunction in Obstructive Sleep Apnea: The Hidden Role of Vitamin D Deficiency
Source: J Evid Based Med. 2025 Sep 17;18(3):e70071. doi: 10.1111/jebm.70071 (PMC12506955; doi:10.1111/jebm.70071)
Supplement: Supplementary file 1 — Figure S1: The recruitment of patients. Table S1: Mean ± SD and range of short‐ and long‐term HRV parameters summarized from available cross‐sectional studies data. Table S2: Parasympathetic nervous system and sympathetic nervous system zones interpretation and stress index normal range (from Kubios user guide). Table S3: Characteristics of study participants. Table S4: Correlation between serum 25(OH)D and heart rate variability parameters. Table S5: Multivariate analysis between heart rate variability parameters and 25(OH)D and covariates in patients with OSA. [file JEBM-18-0-s001.docx]

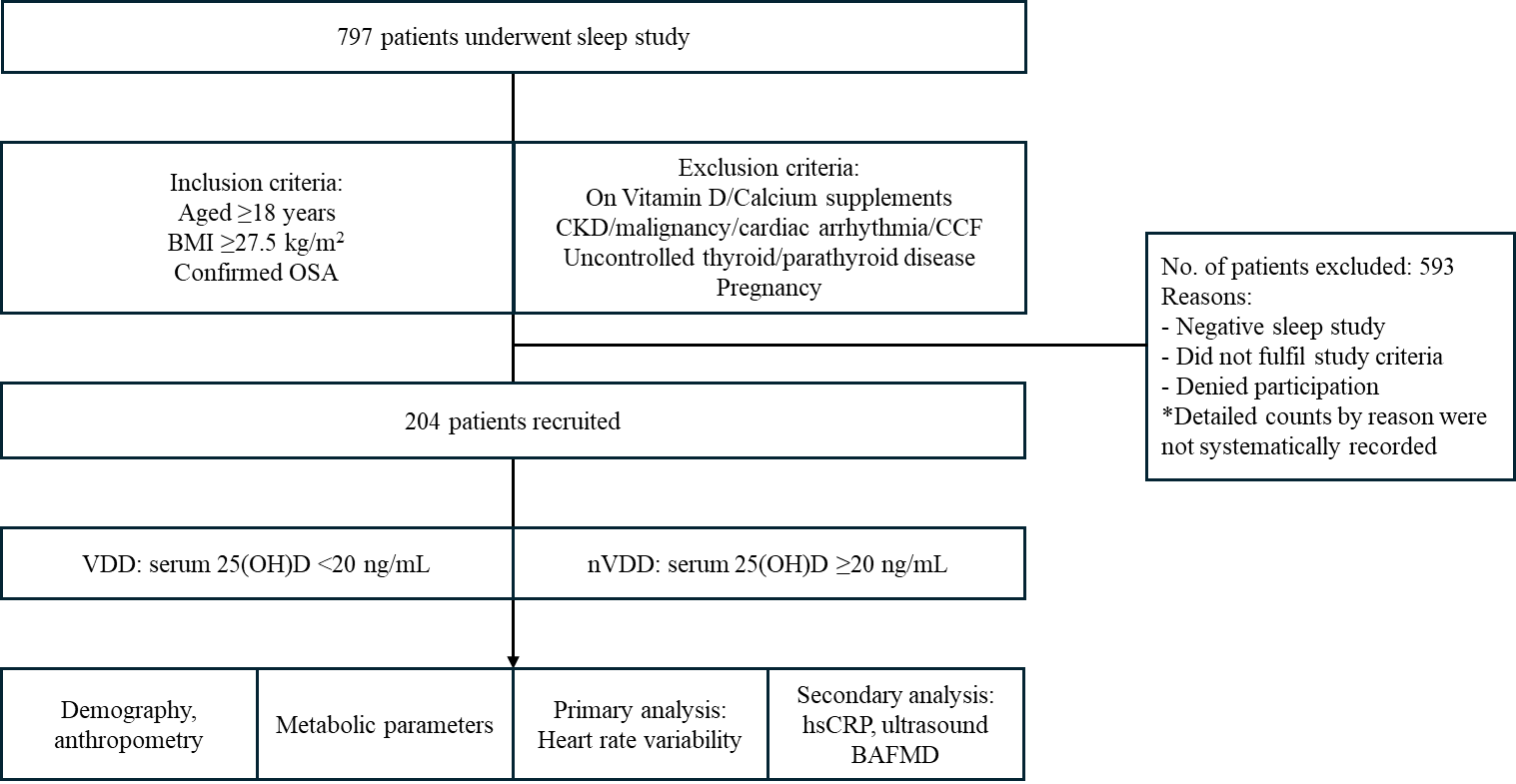


BMI: body mass index; OSA: obstructive sleep apnea; CKD: chronic kidney disease; CCF: congestive cardiac failure; VDD: vitamin D deficiency; 25(OH)D: 25-hydroxyvitamin D; nVDD: non-vitamin D deficiency; hsCRP: high-sensitivity C-reactive protein; BAFMD: brachial artery flow-mediated dilatation

**Supplementary Figure 1 The recruitment of patients**

**Supplementary Table 1**: Mean ± SD and range of short- and long-term HRV parameters summarized from available cross-sectional studies data

| *HRV measures* | *Normal values/ranges* |
| --- | --- |
| *Short-term* |  |
| SDNN | 32-93 |
| Mean RR | 785-1160 |
| RMSSD | 19-75 |
| Stress index | 7-12 |
|  |  |
| *Long-term* |  |
| SDNN | 141±39 |
| SDANN | 127±35 |

SDNN: standard deviation of the NN intervals; RMSSD: square root of the mean of the squares of successive NN interval differences; SDANN: standard deviation of 5-minute average NN intervals

**Supplementary Table 2**: Parasympathetic nervous system and sympathetic nervous system zones interpretation and stress index normal range (from Kubios user guide)

| *PNS zone* | *Interpretation* | *SNS zone* | *Interpretation* |
| --- | --- | --- | --- |
| <-2 | Very low | >2 | Very high |
| -2 to -1 | Low | 1 to 2 | High |
| -1 to 1 | Normal | -1 to 1 | Normal |
| 1 to 2 | High | -2 to -1 | Low |
| >2 | Very high | <-2 | Very low |
|  | | Normal range | |
| Stress index | | 7 to 12 | |
| Log_10_ stress index | | 0.85 to 1.08 | |

**Supplementary Table 3** Characteristics of study participants

|  | All, n=204 | VDD, n=118 | nVDD, n=86 | p |
| --- | --- | --- | --- | --- |
| Age, years | 43.4±12.3 | 41.6±11.7 | 45.8±12.7 | 0.016 |
| Female gender, n (%) | 104 (51.0) | 79 (66.9) | 25 (29.1) | <0.001 |
| Co-morbidities  T2DM  Dyslipidemia  Hypertension  Hyperuricemia | 107 (52.5)  96 (47.1)  160 (78.4)  122 (59.8) | 59 (50.0)  45 (31.1)  90 (76.3)  73 (61.9) | 48 (55.8)  51 (59.3)  70 (81.4)  49 (57.0) | 0.412  0.003  0.380  0.482 |
| Past CAD | 10 (4.9) | 4 (3.4) | 6 (7.0) | 0.199 |
| Smoking  Active smoker  Non-smoker | 38 (18.6)  166 (81.4) | 13 (11.0)  105 (89.0) | 25 (29.1)  61 (70.9) | 0.001 |
| Alcohol consumption  Yes  No | 49 (24.0)  155 (76.0) | 29 (24.6)  89 (75.4) | 20 (23.3)  66 (76.7) | 0.827 |
| AHI, per hour | 39.0 (22.3, 66.0) | 40.1 (19.6, 63.8) | 38.0 (24.5, 67.5) | 0.583 |
| OSA severity  Mild-Moderate  Severe | 76 (37.3)  128 (62.7) | 46 (39.0)  72 (61.0) | 30 (34.9)  56 (65.1) | 0.550 |
| Systolic BP, mmHg | 147.0±19.2 | 146.3±19.5 | 147.9±18.9 | 0.553 |
| Diastolic BP, mmHg | 93.9±13.5 | 93.8±13.7 | 80.6±14.5 | 0.956 |
| BMI, kg/m^2^ | 40.7±7.2 | 42.0±7.1 | 38.9±7.0 | 0.002 |
| Waist circumference, cm | 118.9±14.0 | 119.4±14.3 | 118.0±13.5 | 0.480 |
| 25(OH)D, ng/mL | 19.2±7.3 | 14.2±3.6 | 26.1±5.0 | <0.001 |
| iPTH, mmol/L | 61.9 (43.1, 89.3) | 63.6 (44.2, 93.6) | 60.9 (40.0, 82.5) | 0.167 |
| Corrected calcium, mmol/L | 2.33±0.10 | 2.33±0.10 | 2.32±0.11 | 0.474 |
| Phosphate, mmol/L | 1.19±0.21 | 1.20±0.19 | 1.17±0.23 | 0.307 |
| UACR, mg/mmol | 2.75 (0.90, 8.63) | 3.20 (0.90, 10.10) | 2.10 (0.90, 5.93) | 0.206 |
| Uric acid, mmol/L | 416.2±90.4 | 413.8±90.6 | 419.4±90.5 | 0.810 |
| HbA1c | 6.20 (5.80, 6.8) | 6.20 (5.80, 6.90) | 6.20 (5.70, 6.8) | 0.680 |
| Lipid profile, mmol/L  Total  LDL-C  HDL-C  Triglycerides | 4.78±1.04  2.78±0.84  1.28±0.25  1.54 (1.09, 2.08) | 4.88±1.11  2.84±0.89  1.30±0.27  1.65 (1.11, 2.28) | 4.64±0.93  2.74±0.76  1.27±0.22  1.36 (1.01, 1.93) | 0.113  0.402  0.289  0.050 |
| hsCRP | 4.60 (2.40, 8.78) | 5.60 (2.88, 11.38) | 3.45 (2.0, 7.13) | 0.010 |
| BAFMD ultrasound, % | 6.12±1.45 | 6.27±1.52 | 5.91±1.33 | 0.115 |

Numerical variables are presented as the mean ± standard deviation or median (IQR), categorical variables are defined as percentage.

VDD: vitamin D deficiency; nVDD: non-vitamin D deficiency; T2DM: type 2 diabetes mellitus; CAD: coronary artery disease; AHI: apnoea hypopnea index; OSA: obstructive sleep apnea; BP: blood pressure; BMI: body mass index; 25(OH)D: 25-hydroxyvitamin D; iPTH: intact parathyroid hormone; UACR: urine albumin creatinine ratio; hsCRP: high-sensitivity C-reactive protein; BAFMD: brachial artery flow-mediated dilatation; ASDNN: mean of the standard deviations of all NN intervals for all 5-minute segments of entire recording; SDANN: standard deviation of 5-minute average NN intervals; SDNN: standard deviation of the NN intervals; RMSSD: square root of the mean of the squares of successive NN interval differences; PNS: parasympathetic nervous system; SNS: sympathetic nervous system

**Supplementary Table 4** Correlation between serum 25(OH)D and heart rate variability parameters

*Pearson’s correlation

|  | r | p |
| --- | --- | --- |
| ASDNN | 0.071 | 0.314 |
| SDANN | 0.129 | 0.068 |
| SDNN | 0.119 | 0.092 |
| SDNN (Polar H10) | -0.012 | 0.871 |
| RMMSD | -0.008 | 0.905 |
| PNS index | 0.106 | 0.134 |
| SNS index | -0.185 | 0.009 |
| Stress index | -0.145 | 0.041 |

25(OH)D: 25-hydroxyvitamin D; ASDNN: mean of the standard deviations of all NN intervals for all 5-minute segments of entire recording; SDANN: standard deviation of 5-minute average NN intervals; SDNN: standard deviation of the NN intervals; RMSSD: square root of the mean of the squares of successive NN interval differences; PNS: parasympathetic nervous system; SNS: sympathetic nervous system

**Supplementary Table 5** Multivariate analysis between heart rate variability parameters and 25(OH)D and covariates in patients with OSA

| Independent variable | Dependent variable: SNS index^a^  Adjusted R^2^ = 0.135 | | |
| --- | --- | --- | --- |
|  | β | 95% CI | p |
| Age | -0.034 | -0.067, 0 | 0.051 |
| Smoking | 0.516 | -0.132, 1.164 | 0.118 |
| AHI | 0.019 | 0.004, 0.035 | 0.011 |
| Diastolic BP | 0.028 | -0.002, 0.057 | 0.068 |
| HbA1c | 0.215 | -0.050, 0.480 | 0.111 |
| HDL-C | -1.951 | -3.639, -0.263 | 0.024 |
| 25(OH)D | -0.066 | -0.122, -0.011 | 0.020 |
| Independent variable | Dependent variable: Stress index^b^  Adjusted R^2^ = 0.148 | | |
|  | β | 95% CI | p |
| Age | -0.048 | -0.210, 0.114 | 0.559 |
| Smoking | 1.686 | -1.258, 4.631 | 0.260 |
| AHI | 0.094 | 0.026, 0.163 | 0.007 |
| Diastolic BP | 0.088 | -0.049, 0.225 | 0.206 |
| HbA1c | 0.968 | -0.278, 2.214 | 0.127 |
| LDL-C | -0.895 | -3.567, 1.777 | 0.509 |
| HDL-C | -7.624 | -15.903, 0.655 | 0.071 |
| Triglycerides | -0.603 | -2.961, 1.756 | 0.615 |
| Uric acid | 0.010 | -0.011, 0.031 | 0.364 |
| 25(OH)D | -0.273 | -0.527, -0.020 | 0.034 |

^a^Adjusted for gender, alcohol consumption, BMI, systolic BP, LDL-C, triglyceride level, uric acid level

^b^Adjusted for gender, alcohol consumption, BMI, systolic BP

25(OH)D: 25-hydroxyvitamin D; OSA: obstructive sleep apnea; SNS: sympathetic nervous system; AHI: apnoea hypopnea index; BP: blood pressure; BMI: body mass index
